# COVID-19 and Diabetic Ketoacidosis: Report of Eight Cases

**DOI:** 10.7759/cureus.14223

**Published:** 2021-03-31

**Authors:** Balraj Singh, Prem Patel, Parminder Kaur, Nicole Majachani, Michael Maroules

**Affiliations:** 1 Hematology/Oncology, Saint Joseph's University Medical Center, Paterson, USA; 2 Cardiology, Saint Joseph's University Medical Center, Paterson, USA; 3 Internal Medicine, Saint Joseph's University Medical Center, Paterson, USA; 4 Hematology and Oncology, Saint Joseph's University Medical Center, Paterson, USA

**Keywords:** type i diabetes mellitus, covid 19, diabetic ketoacidosis (dka), ketotic hyperglycemia, types 2 diabetes, sar-cov 2 infection, diabetic hyperglycemic hyperosmolar syndrome

## Abstract

Aim

To describe the clinical characteristics and outcome of hospitalized COVID-19 patients with diabetic ketoacidosis (DKA).

Methods

We report eight cases of diabetic ketoacidosis in COVID-19 who presented to our institution in New Jersey, USA. COVID-19 was diagnosed by nasopharyngeal swab reverse transcription polymerase chain reaction (RT-PCR). The patients' electronic medical records were reviewed. Data on patients' age, sex, ethnicity, laboratory values, glycosylated hemoglobin level, oral antihyperglycemic agents (OHAs), insulin, and clinical outcomes were collected.

Results

The median age of the patient was 42.5 years, and seven were males and one was female. Out of eight patients, five had type 2 diabetes mellitus (DM), two had undiagnosed DM, and one had type 1 DM. Median value of initial glucose on presentation was 454 mg/dL. Median value of HbA1c on presentation was 11.4% and of anion gap was 26.5 mEq/L. Four patients had large ketonemia, one patient had moderate ketonemia, and three patients had small ketonemia. All the patients were started on standard treatment protocol for DKA with intravenous fluids and IV insulin infusion. Acute kidney injury (AKI) was seen in four patients, and one patient required renal replacement therapy. Out of eight patients, three required mechanical ventilation, and the same three patients died.

Conclusion

Our case series shows that COVID-19 infection can precipitate DKA in patients with known diabetes mellitus patients or as a first manifestation in undiagnosed DM patients; COVID-19 with DKA is associated with substantial mortality. Further studies are needed to characterize poor risk factors associated with mortality in these patients.

## Introduction

The severe acute respiratory syndrome coronavirus 2 (SARS-CoV-2) is an enveloped novel RNA beta-coronavirus responsible for the current global pandemic. It has led to a health crisis with rapidly increasing number of cases and fatalities. Although respiratory symptoms predominate, widespread organ-specific manifestations (gastrointestinal, neurological, thromboembolic, immunological, and cardiovascular) of COVID-19 are increasingly being reported. In-depth understanding of various systemic manifestations and complications of SARS-CoV2 is of paramount importance for optimal management of these patients.

As research into COVID-19 expands, knowledge of the endocrine effects is becoming better understood. Diabetes and obesity are common comorbidities associated with COVID-19 [1]. Comorbidities like cardiovascular disease, diabetes mellitus (DM), hypertension, chronic lung disease, cancer, chronic kidney disease, obesity, and smoking have been associated with severe illness and mortality [2,3]. A retrospective study from China found that high fasting glucose levels ≥ 126 mg/dl at admission was an independent poor prognostic factor in patients with COVID-19 who did not have diabetes mellitus [4]. A wide range of manifestations and outcome can occur depending upon the comorbidities of the infected patient. COVID-19 can precipitate metabolic complications of diabetes such as diabetic ketoacidosis (DKA) and hyperosmolar hyperglycemic state (HHS) [5].

## Materials and methods

We report eight cases of diabetic ketoacidosis in COVID-19 who presented to our institution in New Jersey, USA. COVID-19 was confirmed by real-time reverse transcription polymerase chain reaction (RT-PCR) assay. Diabetes was defined as glycated hemoglobin (HbA1c) ≥ 6.5% or any established diagnosis prior admission. DKA is defined by the triad of hyperglycemia, anion gap metabolic acidosis, and ketonemia or ketonuria. The patients' electronic medical records were reviewed. Data on patients' age, sex, ethnicity, laboratory values, glycosylated hemoglobin (HbA1c) level, oral antihyperglycemic agents (OHAs), insulin, and clinical outcomes were collected.

## Results

Pertinent clinical characteristics and laboratory values are summarized in Table 1.

**Table 1:** Summary of pertinent clinical characteristics and laboratory values. Reference ranges are as follows: Hemoglobin A1c 4%-6%, pH 7.36-7.44, bicarbonate 21-31 mEq/L, glucose 70-110 mg/dL, anion gap 3-10 mEq/L, effective osmolarity 283-299 mOsm/kg, white cell count 4.5-11 K/mm^3^, hemoglobin 12-16 g/dL, platelets 140-440 K/mm^3^, troponin less than 0.03 ng/ml, sodium 135-145 mEq/L, potassium 3.5-5 mEq/L, chloride 98-107 mEq/L, phosphorus 2.5-5 mg/dL, BUN 7-23 mg/dL, creatinine 0.6-1.30 mg/dL, AST 13-39 U/L, ALT 7-52 U/L, ESR 0-10 mm/hr, CRP less than 10 mg/L, ferritin 12-300 ng/ml, LDH 140-271 U/L, d-dimer less than 0.5 (mcg/mL), fibrinogen 183-503 mg/dl. M, Male; F, female; DM, diabetes; undiagnosed, first time diagnosed with diabetes; HTN, hypertension; DLD, dyslipidemia; BPH, benign prostatic hyperplasia; SOB, shortness of breath; NR, not reported; NA, not applicable; DPP4, dipeptidyl peptidase 4 inhibitor; SGLT2 inhibitor, sodium glucose co-transporter 2 inhibitor; BMI, body mass index; ND, not done; BUN, blood urea nitrogen; AST, aspartate transaminase; ALT, alanine transaminase; ESR, erythrocyte sedimentation rate; CRP, C-reactive protein; LDH, lactate dehydrogenase; IV, intravenous; SC, subcutaneous; HCQ, hydroxychloroquine.

Variable	Case 1	Case 2	Case 3	Case 4	Case 5	Case 6	Case 7	Case 8
Age/Sex	35/M	50/M	33/M	52/F	32/M	63/M	30/M	66/M
Ethnicity	African American	Hispanic	Hispanic	Hispanic	Hispanic	Bengali	Hispanic	Hispanic
Medical history	DM	HTN, DM, DLD	None	HTN, DM, DLD, asthma, anxiety	DM	HTN, DM, BPH	HTN	HTN, DM, DLD, asthma
Type 1 DM or type 2 DM or undiagnosed	Type 1 DM	Type 2 DM	Undiagnosed	Type 2 DM	Type 2 DM	Type 2 DM	Undiagnosed	Type 2 DM
Presenting signs and symptoms	Generalized weakness, fatigue	SOB, cough, and chills	SOB, fever	SOB, dizziness	SOB, fever	SOB, vomiting, nausea, abdominal chest pain	SOB, fever, chills, hematemesis, hematochezia	SOB, bloody diarrhea, nausea
Home DM medications	Insulin	Insulin and metformin	NA	Insulin, metformin, DPP4 inhibitor, and SGLT2 inhibitor	Metformin and sulfonylureas	NR	NA	Metformin
BMI	22	24.09	41.88	27.92	25.65	26.7	55.1	33.83
HbA1c (%)	17	15.2	11.2	11.6	12.1	11.2	10.5	10
pH	7.1	7.0	7.1	7.1	7.2	7.3	7.3	7.3
Bicarbonate (mEq/L)	6	7	14	16	13	10	12	16
Glucose on presentation (mg/dL)	553	350	698	396	368	404	516	504
Serum ketones	Large	Large	Small	Moderate	Large	Small	Large	Small
Anion Gap (mEq/L)	28	28	27	14	24	32	26	22
Effective osmolarity (mOsm/Kg)	280.7	281	294.78	298.00	284.44	294.44	298.67	282.00
White cell count (K/mm^3^)	9.1	11.6	13.4	23	10.3	7.1	9.4	12.6
Hemoglobin (g/dL)	12.7	14.8	14.3	14.1	14.9	14.5	11.9	7.6
Platelets (K/mm^3^)	133	298	291	245	223	230	344	155
Troponin (ng/mL)	<0.010	0.036	3.84	0.153	0.01	0.01	0.01	2.293
Sodium (mEq/L)	125	131	128	138	132	136	135	127
Potassium (mEq/L)	4.8	5.1	5.6	3.8	3.9	4.0	4.1	4.9
Chloride (mEq/L)	91	96	87	108	95	94	97	89
Phosphorous (mg/dL)	4.8	ND	6.3	2.6	<1.0	3.1	4.5	3.9
BUN (mg/dL)	26	17	33	3.8	13	22	6	80
Creatinine (mg/dL)	1.50	1.18	2.39	1.89	1.01	0.98	0.50	1.80
AST (U/L)	15	14	294	72	30	29	22	227
ALT (U/L)	21	15	151	58	35	25	58	120
ESR (mm/hr)	131	76	29	32	90	73	83	42
CRP (mg/L)	243.7	293.6	271.4	199.8	214	70.6	363.2	233.9
Ferritin (ng/mL)	371	651	3,546	659	1,339	1,434	1,452	3,511
LDH (U/L)	340	327	1,479	510	289	281	527	726
D-dimer (mcg/mL)	ND	1.37	20	19.14	0.74	2.24	0.94	9.34
Fibrinogen (mg/dl)	ND	651	631	811	ND	ND	798	ND
Insulin IV or SC	IV	IV	IV	IV	IV	IV	IV	IV
Acute renal replacement therapy	None	None	Hemodialysis	None	None	None	None	None
Intubated	No	No	Yes	Yes	No	No	No	Yes
Treatment of COVID-19	HCQ, doxycycline	HCQ, azithromycin	Cefepime, azithromycin, vancomycin	HCQ, ceftriaxone, azithromycin	HCQ, azithromycin	HCQ, azithromycin	HCQ, azithromycin	HCQ, ceftriaxone, azithromycin
Outcome	Discharged	Discharged	Died	Died	Discharged	Discharged	Discharged	Died

The median age of the patient was 42.5 years (range 30-66 years), and seven were males and one was female. Ethnicity distribution in our patients: sex were Hispanic, one was African American, and one was Bengali. Comorbidities of the patients were hypertension, dyslipidemia, asthma, anxiety, and benign prostatic hyperplasia. Out of eight patients, five had type 2 DM, two were undiagnosed with DM, and one had type 1 DM. Presenting complaints included generalized weakness, fatigue, SOB, cough, fever, chills, dizziness, nausea, vomiting, abdominal pain, chest pain, and blood in stools. One patient was taking SGLT 2 inhibitors (known to increase the risk of ketoacidosis). Median value of initial glucose on presentation was 454 mg/dL. Median value of HbA1c on presentation was 11.4% and of anion gap was 26.5 mEq/L. Four patients had large ketonemia, one patient had moderate ketonemia, and three patients had small ketonemia. All the patients were started on standard treatment protocol for DKA with intravenous fluids and IV insulin infusion. Inflammatory markers (CRP, ferritin) were elevated in all the patients. Acute kidney injury (AKI) was seen in four patients, and one patient required renal replacement therapy. Out of eight patients, three required mechanical ventilation, and the same three patients died.

## Discussion

It is estimated that in 2018, in the United States, about 10.5% of the population had diabetes, while 21.4% of the patients that have diabetes are unaware. Diabetes is known to cause both microvascular and macrovascular complications, and patients with diabetes have multiple coexisting conditions and complications. In 2017, the total direct and indirect estimated cost of diagnosed DM in the United States was $327 billion. In the last three decades, the number of patients with DM has quadrupled with specific races and ethnicities having disproportionate increases compared to others. With lifestyle changes, diabetes mellitus-related complications can be prevented [6].

Diabetic ketoacidosis is a potentially lethal complication of DM, often occurring more frequently in patients with DM type I. Observations during the COVID-19 pandemic have noted that patients with pre-existing diabetes may be at increased risk of DKA in the setting of COVID-19 infection [7]. Early data from Italy showed that 35.5% of patients that passed away with COVID-19 had coexisting diabetes [8]. Boddu et al. also evaluated the association between COVID-19-induced diabetes and pointed out a bidirectional link between COVID-19 and type 1 DM; though there is a hypothesis of new onset type I DM caused by COVID-19, no conclusive link has been made [9]. Further long-term studies that follow these patients will help us fully understand the connection between diabetes and COVID-19.

In the setting of the COVID-19 pandemic, unique considerations need to be applied to the management of DKA. The use of corticosteroids, specifically dexamethasone, has shown improved outcomes in patients hospitalized and requiring oxygen support [10]. With the use of corticosteroids for COVID-19 management, careful monitoring is required for hyperglycemia. Appropriate glycemic control and cessation of ketone production with insulin and correction of the fluid and electrolyte abnormalities are the cornerstone of DKA management; however, this requires extensive bedside patient interaction to monitor blood glucose, administer and titrate medications, and monitor rapid interventions for hypoglycemic events [11]. Though intravenous insulin has been the gold standard for management of DKA, there are unique considerations in the setting of COVID-19 pandemic as minimizing exposure to healthcare workers at bedside and preserving personal protective equipment become more important. In the setting of mild to moderate uncomplicated DKA, subcutaneous rapid-acting insulin every one to two hours is a safe and effective alternative [12].

Rubino et al. proposed that the virus, in binding to angiotensin-converting enzyme 2 (ACE2) receptors that are present in the beta cells of the pancreas, may cause pleiotropic alterations in glucose metabolism, which may ultimately exacerbate pre-existing diabetes or may even lead to a new mechanism of manifestation of disease [13]. Initial concerns originating from animal studies proposed that ACE2 expression may be upregulated in patients taking ACE inhibitors and angiotensin receptor blockers. These medications are frequently taken by diabetic patients for comorbidities. However, three subsequent studies, on various populations, did not provide evidence to support this hypothesis [14].

Most recent literature shows that diabetic patients with COVID-19 have an increased mortality. Early studies in April 2020 that consist of 5,700 patients hospitalized with COVID-19 in the New York city area showed that patients with diabetes were more likely to be have received invasive mechanical ventilation [1]. Guo et al. reported higher levels of inflammation-related biomarkers in DM patients compared to non-DM patients and DM as a risk factor for the progression and prognosis of COVID‐19 patients [15]. A retrospective cross-sectional study in COVID-19-infected patients showed that patients with diabetes were more likely to have longer length of stay (LOS) in hospital (14.4 days) as compared to the patients without diabetes (9.8 days) [16].

## Conclusions

The case series we presented highlights there are great challenges in the co-management of COVID-19 and DKA. COVID-19 may precipitate DKA in patients with a history of DM or may even occur in patients with undiagnosed diabetes. In the setting of COVID-19 and DKA, patients who are mechanically ventilated do have a poorer outcome. Furthermore, DM patients have higher inflammation-related markers and longer LOS as compared to non-DM patients. Further studies are needed to address the pathophysiology and risk factors in order to understand better treatment strategies while addressing the unique circumstances of the COVID-19 pandemic. During the COVID-19 pandemic, in patients with DM, tight control of glucose levels and prevention of diabetes-related complications are of paramount importance to prevent severe courses of COVID-19. Healthcare providers should aim to reduce the exposure in vulnerable diabetes population.
